# Supplementary material for: Electrochemical Detection of Ammonia in Water Using NiCu Carbonate Hydroxide-Modified Carbon Cloth Electrodes: A Simple Sensing Method
Source: Sensors (Basel). 2024 Jul 25;24(15):4824. doi: 10.3390/s24154824 (PMC11315050; doi:10.3390/s24154824)
Supplement: Supplementary file 1 [file sensors-24-04824-s001.zip › sensors-3106320-supplementary.pdf]

Table S1. Experimental Reagents.

Chemical Formula	Reagent Grade	Manufacturer
KOH	Analytical Reagent (AR)	China National Pharmaceutical Group Chemical Reagent Co., Ltd.
NaCl	AR	China National Pharmaceutical Group Chemical Reagent Co., Ltd.
NH_4F	AR	Xilong Scientific Co., Ltd.
Na_2CO_3	AR	Xilong Scientific Co., Ltd.
KCl	AR	Xilong Scientific Co., Ltd.
$Cu(NO_3)_2 \cdot 3H_2O$	AR	Xilong Scientific Co., Ltd.
$CO(NH_2)_2$	AR	Xilong Scientific Co., Ltd.
$K_3[Fe(CN)_6]$	AR	Beijing Bei Hua Fine Chemicals Co., Ltd.
$NaNO_3$	AR	Beijing Bei Hua Fine Chemicals Co., Ltd.
Na_2SO_4	AR	Beijing Bei Hua Fine Chemicals Co., Ltd.
NH_4Cl	AR	Beijing Bei Hua Fine Chemicals Co., Ltd.
$Ni(NO_3)_2 \cdot 6H_2O$	AR	Tianjin Yong Sheng Fine Chemicals Co., Ltd.

Table S2. Experimental Instruments.

Instrument Name	Model	Manufacturer
Magnetic Stirrer	MS-500	INTLLAB Laboratory Equipment
Ultrasonic Cleaner	SN-QX-13	Zhejiang Lichen Instrument Co., Ltd.
Field Emission Scanning Electron Microscope (SEM)	JEOL-JEM-6700F	JEOL Ltd.
X-ray Photoelectron Spectrometer (XPS)	EXCARA-250	Thermo Fisher Scientific Inc.
X-ray Diffractometer (XRD)	Shimadzu 6000	Shimadzu Corporation
Electrochemical Workstation	CHI760D	Shanghai Chenhua Instrument Co., Ltd.

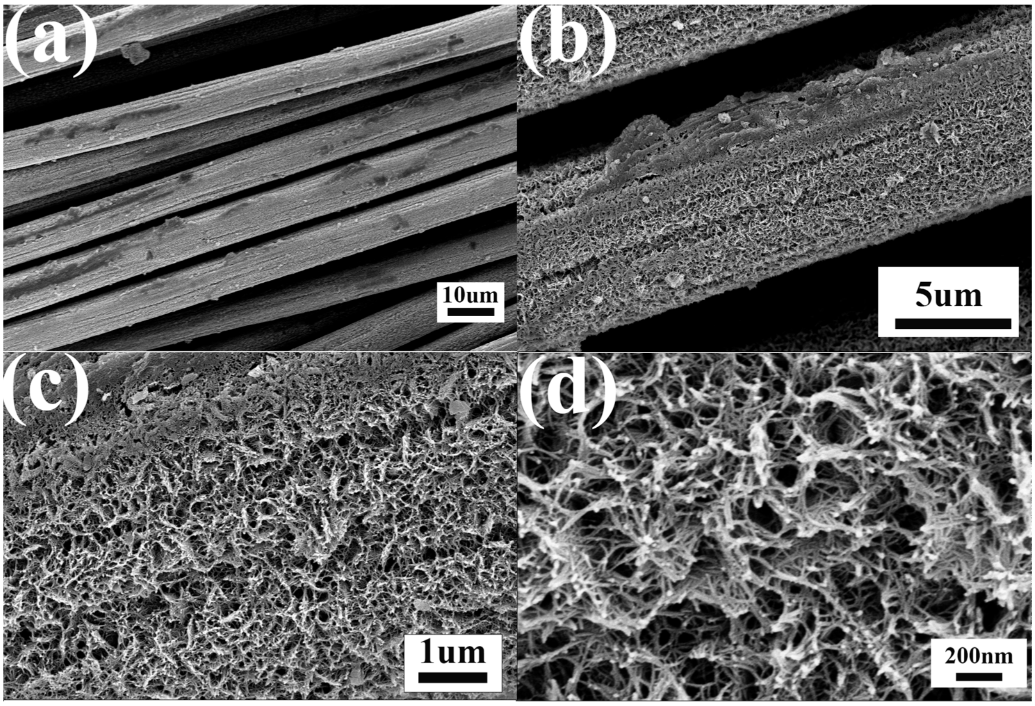

Figure 1. SEM images of NC-10@CC.

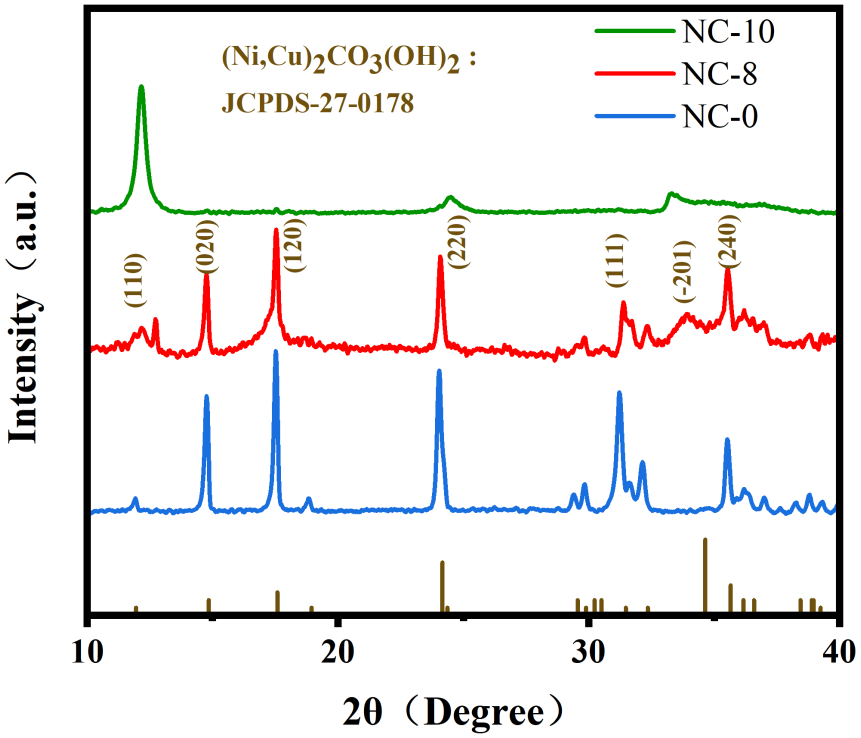

Figure S2. XRD pattern of the powder material synthesized by hydrothermal precipitation.

Table S3. ECSA and Rct of all electrodes.

**electrode**	**ECSA(cm^2)**	**Rct(Ω)**
CC	2.853	27.3
NC-8@CC	1.66	105
NC-0@CC	1.452	139.8
NC-10@CC	1.064	153.5
